# Hydrothermally extraction of saponin from *Acanthophyllum glandulosum* root – Physico-chemical characteristics and antibacterial activity evaluation

**DOI:** 10.1016/j.btre.2020.e00507

**Published:** 2020-07-23

**Authors:** Roza Najjar-Tabrizi, Afshin Javadi, Anousheh Sharifan, Kit Wayne Chew, Chyi-How Lay, Pau Loke Show, Hoda Jafarizadeh-Malmiri, Aydin Berenjian

**Affiliations:** a Department of Food Science and Technology, Science and Research Branch, Islamic Azad University, Tehran, Iran; b Department of Food Hygiene, Tabriz Branch, Islamic Azad University, Tabriz, Iran; c School of Energy and Chemical Engineering, Xiamen University Malaysia, Jalan Sunsuria, Bandar Sunsuria, 43900 Sepang, Selangor Darul Ehsan, Malaysia; d Master's Program of Green Energy Science and Technology, Feng Chia University, No. 100, Wenhwa Road, Seatwen, Taichung 40724, Taiwan; e General Education Center, Feng Chia University, No. 100, Wenhwa Road, Seatwen, Taichung 40724, Taiwan; f Green Energy and Biotechnology Industry Research Center, Feng Chia University, No. 100, Wenhwa Road, Seatwen, Taichung 40724, Taiwan; g Department of Chemical and Environmental Engineering, Faculty of Science and Engineering, University of Nottingham Malaysia, Jalan Broga, 43500 Semenyih, Selangor Darul Ehsan, Malaysia; h Faculty of Chemical Engineering, Sahand University of Technology, Tabriz 51335-1996, East Azarbaijan, Iran; i School of Engineering, Faculty of Science and Engineering, University of Waikato, Hamilton 3240, New Zealand

**Keywords:** *Acanthophyllum glandulosum*root, Antibacterial activity, Antioxidant activity, Hydrothermal extraction, Saponin

## Abstract

Saponin was extracted from *Acanthophyllum glandulosum* root under subcritical water conditions, and effects of root powder and pH of the solution were evaluated on the concentration of the saponin as manifested in its foamability and antioxidant activity using RSM. FT-IR analysis indicated that *A. glandulosum* root extract had 2 main functional groups (hydroxyl and amide I groups). Saponin with the highest foam height (4.66 cm), concentration (0.080 ppm) and antioxidant activity (90.6 %) was extracted using 10 g of the root powder and pH value of 4. Non-significant differences were observed between the predicted and experimental values of the extraction response variables. The study demonstrated good appropriateness of resulted models by Response Surface Methodology. Furthermore, higher values of R^2^ was attained for the foamability (>0.81) and antioxidant activity (>0.97), as well as large p-values (p > 0.05) indication of their lack-of-fit response verified the acceptable fitness of the provided models. The extracted saponin also showed bactericidal effect, which shows potential as a natural antibacterial compound.

## Highlights

• Saponin extraction was performed hydrothermally (121 °C and 1.5 atm for 15 min).
• Optimum extraction conditions: 10 g of the root powder and pH of 4.
• Optimum results: foam height (4.66 cm), concentration (0.080 ppm) and antioxidant activity (90.6 %).
• Demonstration of appropriateness of resulted models by RSM.
• Extracted saponin from *A. glandulosum* root had bactericidal effect.

## 1 Introduction

*Acanthophyllum* belongs to *Caryophyllaceae* family with 61 species, in which 33 species are grown in Iran. Most of them are found in the eastern regions of Iran, especially in Khorasan Province, and it is locally known as Chubak [1,2]. Root of *Acanthophyllum* is a rich source of saponin, a natural biosurfactant with high potential applications in food industries. However, saponin is extracted from several natural sources such as *Ziziphus spina-christi*, *Glycyrrhiza glabra* root, plum and strawberry fruits, but *Acanthophyllum glandulosum* root is the primary source of saponin [[3], [4], [5]].

Saponin is known as defense system of the plants toward pathogenic microorganisms and has tri-terpenoids or steroid glycosides which that conjugates to sugar chains (one or more), with glycoside bond in its structure [6,7]. Saponin is a non-ionic natural emulsifier, due to its high surface tension reduction activity between two immiscible fluids which that increase its applications extensively in food, cosmetics, detergents and pharmaceutical industries [[8], [9], [10]]. Furthermore, saponin has hemolytic activity, anti-inflammatory, antifungal and adjuvant properties [11].

Several methods have been commonly utilized to extract valuable compounds from medicinal plants, such as Soxhlet, maceration and reflux extractions which are established on utilizing organic solvents for long heating time [12]. Due to thermal decomposition and oxidation of the main bioactive compounds, such as saponin, via traditionally extraction methods which those decrease the extraction yield, and increase energy consuming and time, due to further processes to remove solvents, new extraction methods based on utilizing microwave, ultrasound and pulsed electric field have been developed [1,10,11]. However, these new techniques decrease the process time and organic solvents consumption and at the same time have suitable efficiency for extracting target compounds, but those need high capital and maintenance costs for applying in the industrial scales [1].

Subcritical water is well-known as pressurized water (pressure of higher than 1 bar) with high temperature (higher than 100 °C) which has its liquid state at the mentioned conditions and its polarity leads into ethanol and methanol and increases the extraction yield [13]. These conditions can be rapidly provided in a laboratory autoclave and can be easily scaled up for industrial applications. Furthermore, changes in the pH, temperature and pressure of the subcritical water can effectively cause plant cell wall disintegration and increase the performance of extraction [1,14]. Therefore, the objectives of this research are to i) evaluate effects of pH and amount of *A. glandulosum* root powder on saponin extraction yield through hydrothermally extraction method, and ii) assess foamability, antioxidant and antibacterial activities of the saponin by obtained optimal extraction conditions.

## 2 Materials and methods

### 2.1 Materials

*A. glandulosum* root (in dried state) was provided from a local traditional market (Tehran, Iran). Saponin was obtained from Merck Company (Merck KGaA, Darmstadt, Germany). Distilled water (DW), as a solvent, was bought from Dr. Mojallali Chemical Complex Co. (Tehran, Iran). *Escherichia coli* (PTCC 1276) and *Staphylococcus aureus* (PTCC 1431) were purchased from the biological source from Persian Type Culture Collection (PTCC, Tehran, Iran). Nutrient agar source was obtained from Biolife (Biolife Co., Milan, Italy).

### 2.2 Saponin extraction

*A. glandulosum* roots were washed, dried and ground by an electrical grinder (MX-GX1521, Panasonic, Tokyo, Japan). Defined amounts of the produced powder ranging 10−20 g, were dissolved into 100 mL DW and the pH of solutions was adjusted between 4–9. Provided solutions were transferred into an autoclave and heated at 121 °C and 1.5 atm, for 15 min. After that, the samples were filtered using No.1 Whatman filter paper and kept at refrigerator for further analysis [10].

### 2.3 Physico-chemical analysis

Chief existed functional groups in the *A. glandulosum* root extract, were monitored using Fourier transform infrared (FT-IR) spectrometer on a Bruker Tensor spectrometer (Ettlingen, Germany) at the 4000–400 cm^−1^ region. Turbidity and colour intensity of the extracted samples which those were qualitatively related to the existing saponin in the aqueous solutions, were assessed using UV–vis spectrophotometer (Stone, UK) at a wavelength of 420 nm and 625 nm, respectively. The absorbance unit (% a.u.) obtained was used to identify the colour and turbidity characteristics of the saponin extracts [14]. To monitor the emulsification properties of the extracted saponin from *A. glandulosum* root extract, the filtered samples were hand-shaken vigorously for 30 s and the volume of the foam generated was measured. High performance liquid chromatography (HPLC) (D-14,163 Knauer, Berline, Germany) with a C 18 column (Eurospher 100−5 c18) and diode-array detector was utilized to measure concentration of saponin in the extracted samples. Wavelength of the instrument was fixed at 203 nm. For this test, extracted samples were added into mobile phase containing acetonitrile (40 % v/v) - water (60 % v/v) and injected into the system with sampling rate of 1 (points/second) and total flow rate of 1 mL/min. All the created peaks ranging 2–15 min (retention time) were recorded [10]. Concentration of the extracted saponin was calculated based on plotted standard curve has been established, which that was generated by serial diluted solutions of pure saponin (0.1, 0.01, 0.001 and 0.0001 ppm) and the concentration of the saponin in the sample was correlated into the obtained peak height centered at specific retention time (Eq. 1).

(1) Area = 10^7^ × X^−0.556^ R^2^ = 0.9336

Antioxidant activity of the extracted saponin, was assessed according to free radical-scavenging manner [15]. For this test, 100 μL of the extracted saponin and 5 mL of 50 % (v/v) methanol having 1 mM DPPH radicals, were mixed vigorously and stored in dark bottles (at 27 °C, for 30 min). Pure DPPH and methanol at a ratio of 1:1 were mixed and utilized as the control sample. Using UV–vis spectrophotometer, the maximum absorbance of the samples (DPPH_abs_) and control (Sample extract _abs_) was measured at wavelength of 517 nm, and antioxidant property of the extracted saponins, based on the percentage of DPPH radical scavenging was obtained by Equation (2):(2)
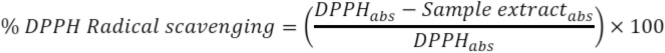


### 2.4 Bactericidal effects of the extracted saponin

Bactericidal activity of the extracted saponin from the provided root powder, toward *S. aureus* and *E. coli* was assessed using well diffusion method. Bacterial suspensions, having 1.5 × 10^8^ colony forming units per mL and based on 0.5 McFarland standard solution, were provided and the surface of set PCA culture media in the plates was inoculated with 0.1 mL of them. Several holes, in 5 mm diameter, were punched in the PCA and 10 μL of the extracted samples were placed into them and incubated at 37 °C, for 24 h. Bactericidal effect of the extracted saponins, was manifested in diameters of inhibition growth zones, around the holes and where, higher diameter, shows higher antibacterial activity and vice versa [16,17].

## 3 Design of experiments and data statistical analysis

Central composite design (CCD) and response surface methodology (RSM) were utilized to experimental design and evaluate of the effects of two independent parameters, namely amount of *A. glandulosum* root powder (X_1_) and pH of the mixture solutions (X_2_), on the foam volume and antioxidant activity of the extracted solutions. RSM has shown many advantages compared to the conventional one-variable-at-a-time method, particularly in generating large amounts of data from a small number of experimental runs. The potential of RSM as a model to analyze the interaction between several variables on the responses makes it a useful technique to evaluate the relationship between the nandispersion preparation variables and response variables of the prepared nanodispersions [16,17]. Furthermore, RSM has minimum number of experiment runs with adequate replications at center point [18].

According to the CCD, 13 experimental runs were achieved with five replications for center point (Table 1) using Minitab software (v.16 statistical package, Minitab Inc., Pennsylvania State, PA, USA). A second order polynomial Eq. (3) having constant (β_0_), linear (β_1_ and β_2_), quadratic (β_11_ and β_22_) and interaction (β_12_) terms, was employed to model response parameters as function of the two independent parameters [[17], [18], [19]].

(3) Y = β_0_ + β_1_X_1_ + β_2_X_2_ + β_11_X_1_^2^ + β_22_X_2_^2^ + β_12_X_1_X_2_

**Table 1:** Central composite design, independent variables' levels and experimental and predicted values of response variables.

Sample No.	Powder weight(g)	pH	Foam height (cm)	Antioxidant activity (%)	Colour (% a.u.)	Turbidity (% a.u.)
1	20	6.5	3.6	90.5	0.708	0.111
2	15	9	4	87	0.488	0.061
3	15	4	3.6	90.6	0.611	0.128
4	11.46	8.2	3.7	*	0.564	0.115
5	10	6.5	3	89.7	0.583	0.106
6	15	6.5	3	89.7	0.463	0.119
7	18.53	4.7	2.7	90.2	0.787	0.129
8	11.46	4.7	3.6	90.3	0.509	0.117
9	15	6.5	2.8	90.1	0.609	0.106
10	18.53	8.2	*	88.8	0.681	0.143
11	15	6.5	2.3	*	0.690	0.119
12	15	6.5	2.7	89.9	0.587	0.141
13	15	6.5	2.7	89.9	0.587	0.141

* Out of range.

Suitability of the models were studied based on the coefficient of determination, R^2^, obtained. The analysis of variance (ANOVA) study was employed to show significance/non-significance of the terms of the generated models according to their P-values (< 0.05) [19].

Surface and contour plots were employed to well observe the extraction factors effects on the dependent factors [20]. Graphical and numerical optimizations were also performed to determine optimum area and exact amounts of *A. glandulosum* root and pH of the solutions to extract saponin with maximum foamability and antioxidant activity. Appropriateness and precision of the produced models were certified by extraction of saponin with attained optimal extraction factors and assessment of the achieved values for the dependent factors, in experimentally and prediction manners [21].

## 4 Results and discussion

### 4.1 Main functional groups existed in *Elaeagnus angustifolia* leaf extract

**Fig. 1:**
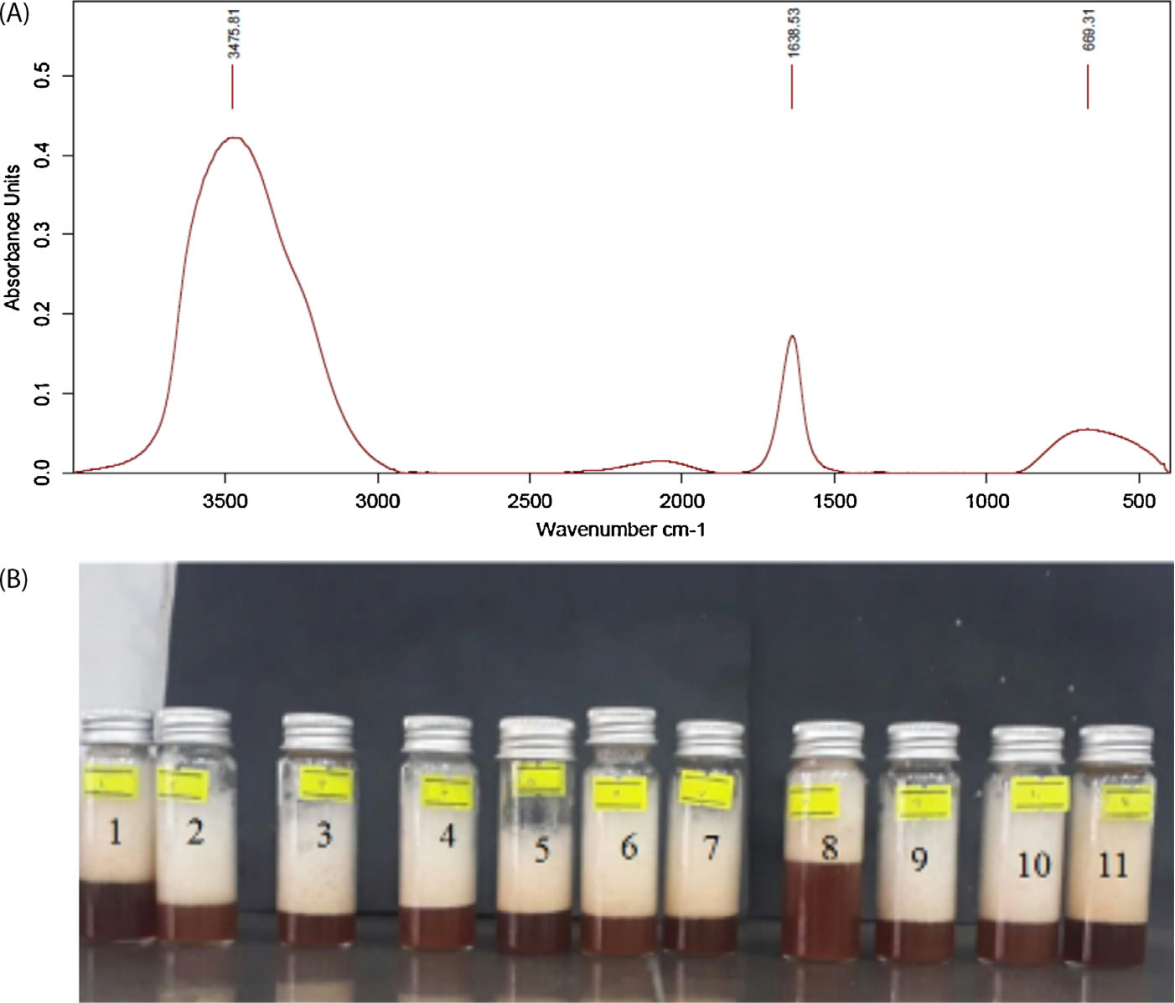
FT-IR spectrum of the extracted saponin (A) and foam height of the extracted solutions containing saponin according to the CCD (B).

FT-IR spectrum of *A. glandulosum* root (Fig. 1A) indicated two main peaks centered at 3475.81 and 1638.53 cm^−1^ which corresponds to the stretching vibration of the −OH and the amide I groups, respectively, which the chemical structure of saponin are composed from these two main groups [14]. Attained results were close to the findings of Mohaddes-Kamranshahi et al. [10]. They demonstrated that the extracted saponin from *Ziziphus spina-christi* leaves had a polyhydroxyl structure containing the amide I group.

### 4.2 Models generation

Based on the design of experiments, the experimental values for the foam heig

ht (foamability), antioxidant activity, colour intensity and turbidity of the extracted samples containing saponin (Table 1) were achieved and according to these values, second polynomial models were generated for the foamability and antioxidant activity of the extracted saponin from *A. glandulosum* root as function of root powder and pH of the solution.

The regression coefficients of the models generated along with their R^2^ and P-values of the lack-of-fit for these two models are showed in Table 2. From the results, the high values of the R^2^ (> 0.81) and (> 0.97) relates to the foamability and antioxidant activity of the extracted saponin, respectively, while the high P-values (P-value > 0.05) of the lack-of-fit for both of them verified the sufficient fitness of the models generated based on the experimental data obtained. As can be observed from the table, linear and quadratic terms of pH had significant (p < 0.05) effects on both the response variables and pH showed a profound effect in extraction of saponin from *A. glandulosum* root. However, only quadratic term of amount of root and interaction term of both selected independent variables had significant effects on foamability and antioxidant activity of the extracted saponin from *A. glandulosum* root, respectively.

**Table 2:** Coefficients of determination R^2^, R^2^-adj, lack of fit (p-value of the regression) and analysis of variance (ANOVA) for the terms of the final model based on p-value and F-ratio.

Coefficients of determination	Foam height (cm)	Antioxidant activity (%)
β_0_(constant)	20.11	90.67
β_1_(main effect)	−1.09	−0.36
P-value	0.028	0.231
β_2_(main effect)	−3.03	1.07
P-value	0.006	0.048
β_11_(quadratic effect)	0.02	0.02
P-value	0.048	0.699
β_22_(quadratic effect)	0.17	−0.19
P-value	0.004	0.002
β_12_(interaction effect)	0.05	0.05
P-value	0.118	0.039
R^2^	0.8125	0.9708
p-value (lack of fit)	0.196	0.135

### 4.3 Effectiveness of independent variables on foamability of the extracted saponin

**Fig. 2:**
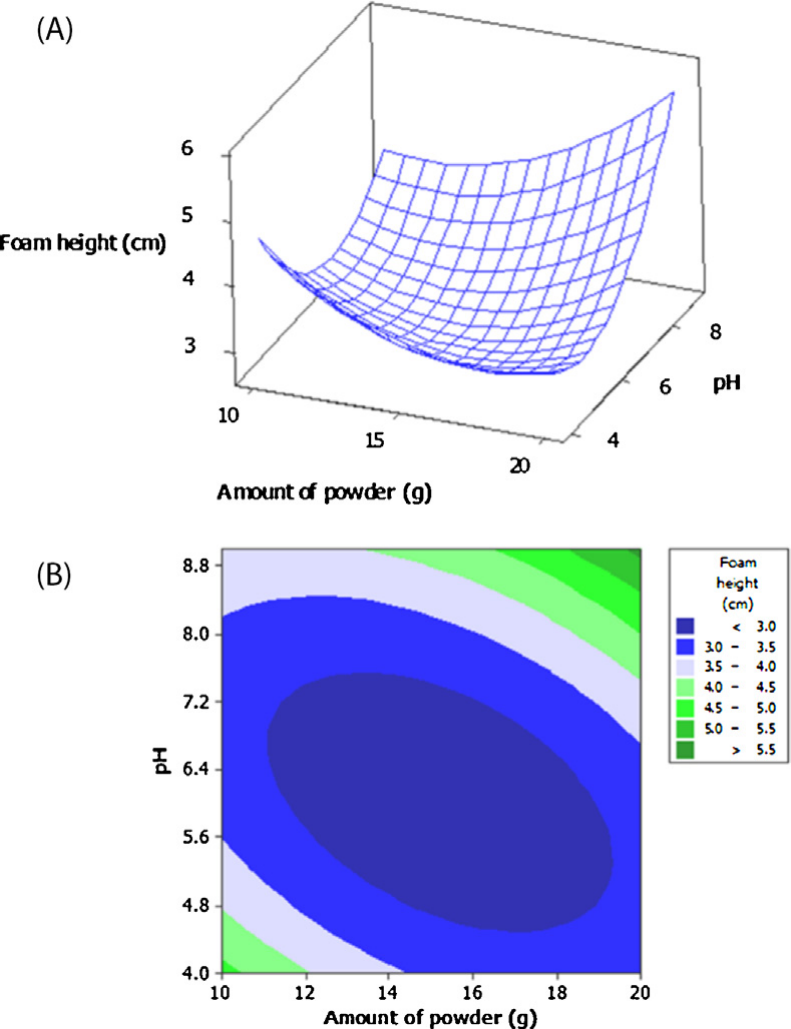
Surface plot (A) and contour plot (B) for foam height (cm) of extracted saponin from *A. glandulosum* root as function of the amount of root powder and pH of the solution.

In Table 1, foam height of the extracted saponin varied from 2.3–4 cm. Fig. 1B shows foam height of the extracted samples according to the CCD (Table 1). In fact, there is a direct relationship between foamability of the extracted solution and its saponin content, where high foamability shows higher amounts of saponin in the extracted solutions and vice versa [10]. Effects of the studied extracted variables on the foamability of the extracted saponin from *A. glandulosum* root is showed in Fig. 2. As clearly observed in Fig. 2A, at a constant value of pH, by raising the content of plant root, foamability of the extracted saponin solution did not change significantly (P < 0.05). However, at constant amount of the root powder, by increasing the pH, foamability also increased significantly (P < 0.05). The absence of curvature in Fig. 2A indicated that interactive term of the both selected variables had insignificant effect on the foamability of the extracted saponin. Our result was verified by higher p-value of the interactive term as can be shown in Table 2.

According to Fig. 2B, highest foamability observed in the solutions which those were extracted using highest amount of plant root powder and pH value, or minimum amount of the plant root and pH value. Obtained results can be related to the point that, by raising the amount of *A. glandulosum* root, saponin content also increased which that causing higher foamability [22]. On the other hand, at strong basic and acidic solutions, surrounded layer made of pectic, cellulosic and hemi cellulosic compounds, which those cover the saponin, were hydrolyzed and maximum amounts of the saponin could be extracted. Attained results were similar to finding from Liu et al. [23]. They also found that maximum amount of saponin was extracted from *Camellia oleifera* at the media with pH of 4.1.

### 4.4 Effectiveness of extraction parameters on antioxidant activity of the saponin

**Fig. 3:**
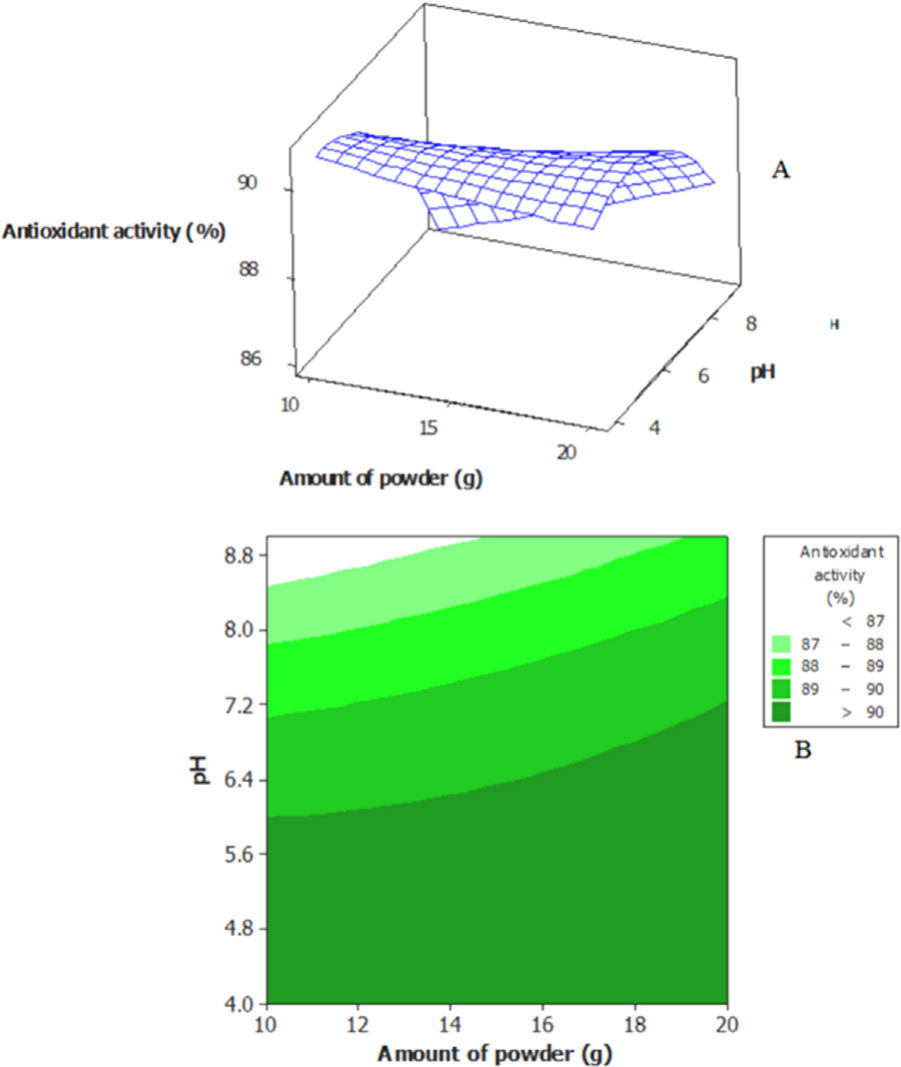
Surface plot (A) and contour plot (B) for antioxidant activity (%) of extracted saponin from *A. glandulosum* root as function of the amount of root powder and pH of the solution.

According to Table 1, antioxidant activity of the extracted saponin solutions varied from 87 to 90.6 %. Fig. 3 shows influences of the selected extraction factors on antioxidant activity of the extracted saponin from *A. glandulosum* root, in which based on the CCD (Table 1). Based on Fig. 3A, at constant and low values of pH, by raising the amount of plant root, antioxidant activity of the extracted saponin solution did not change significantly (P < 0.05). However, at constant and high values of pH, by raising the amount of plant root, antioxidant activity of the extracted saponin solution increased significantly (P < 0.05). Presence of curvature in the Fig. 3A indicated that interaction term of the both selected variables had significant effect (P < 0.05) on antioxidant activity of the extracted saponin. Obtained result was verified by higher P-value of the interactive term as appeared in Table 2. Based on Fig. 3B, maximum antioxidant activity observes in the solutions which those were extracted at acidic solutions (lower pH values).

### 4.5 Optimization of the saponin extraction process from *A. Glandulosum* root

**Fig. 4:**
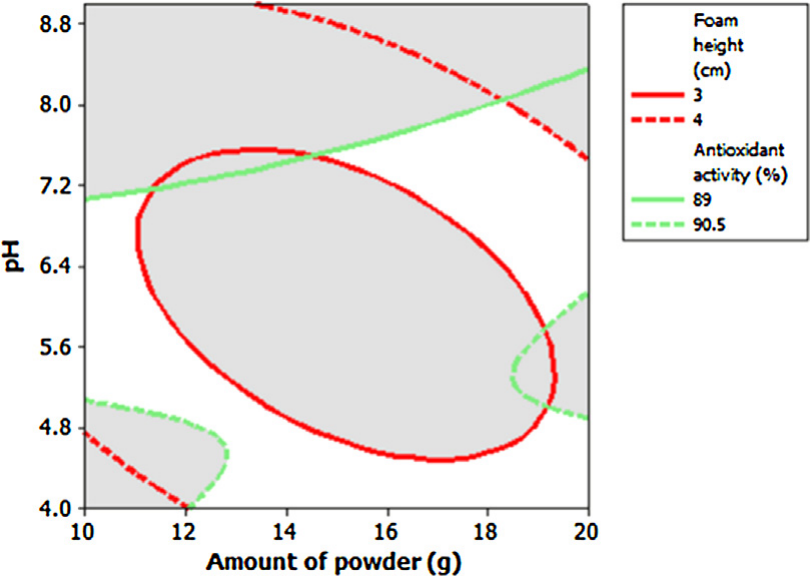
Overlaid contour plot of foam height (cm) and antioxidant activity (%) with acceptable levels as function of the amount of root powder and pH of the solution.

In order to extract saponin from *A. glandulosum* root powder with highest concentration (foamability) and antioxidant activity, obtained numerical optimization result revealed that hydrothermally extraction of saponin using 10 g *A. glandulosum* root powder and pH of 4 for the solution attained to extract saponin with highest foam height, 4.66 cm and antioxidant activity of 90.6 %. Graphical optimization shows optimum area for amounts of both selected independent variables (Fig. 4). Experimental data for the extracted saponin foam height (4.4 ± 0.2 cm) and antioxidant activity (90.5 ± 0.1 %) using obtained optimum extraction factors demonstrated that there was non-significant (P > 0.05) difference between the values of the experimental and predicted response variables of the extracted saponin which in turn, revealed the appropriateness of the generated models by RSM.

### 4.6 Physico-chemical properties of the extracted saponin using obtained optimum extraction parameters

**Fig. 5:**
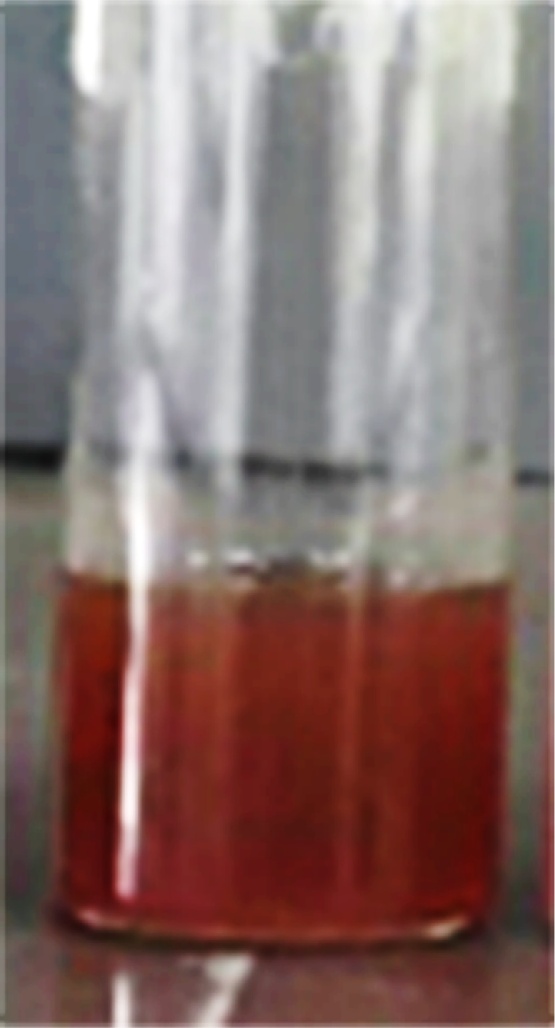
Colour, turbidity and appearance of the extracted saponin from *A. glandulosum* root using obtained optimum extraction parameters.

**Fig. 6:**
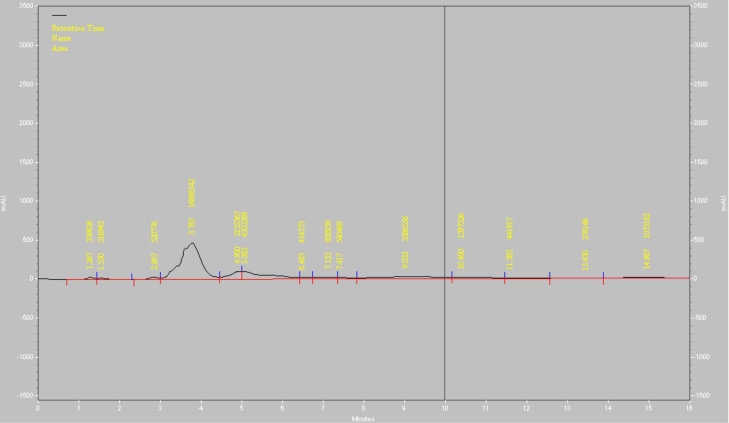
HPLC profile of the extracted saponin from the from *A. glandulosum* root using obtained optimum extraction parameters.

Based on Table 1, colour intensity and turbidity of the extracted solutions containing saponin varied from 0.488 to 0.787 % a.u. and 0.061 to 0.143 % a.u., respectively. Obtained results indicated that extracted saponin from *A. glandulosum* root using obtained optimum extraction conditions had colour intensity and turbidity of 0.595 and 0.110 % a.u., respectively (Fig. 5). Furthermore, concentration of the saponin in the extracted solution from *A. glandulosum* root using optimum values of the independent variables was 0.080 ppm. Fig. 6 shows the HPLC profile of the extracted saponin from *A. glandulosum* root using optimum extraction conditions. The sharp peak centered at 3.7 min (retention time) was related to the extracted saponin.

### 4.7 Antibacterial activity of the extracted saponin from *A. Glandulosum* root

Bactericidal effects of the extracted saponin from *A. glandulosum* root using obtained optimum hydrothermally extraction conditions against *S. aureus* and *E.coli* indicated that its antibacterial activity, manifested as diameter of clear zone, against *S. aureus* (14 mm) was higher than that of on the *E. coli* (11 mm), because of the higher crated clear zone around the wells.

## 5 Conclusions

Saponin, a natural emulsifier, has been utilized in numerous emulsions and nanoemulsions for applications in food and medicine. Extraction of saponin from local and rich natural sources is more interested subject, specially using novel and green extraction methods. Subcritical water, a green solvent, has polarity close to the polarity of methanol, which in turn, increases the extraction yield of saponin from *A. glandulosum* root without need to further chemical solvents and solvent removal process at the end of extraction. In addition to using the simple, environmental friendly, low energy and cost-effective hydrothermal extraction technique based on subcritical water, optimization of other extraction parameters namely amount of *A. glandulosum* root and pH of the solution, could effectively increase extraction yield of saponin and obtained results revealed that using minimum amount of *A. glandulosum* root into the acidic solution, maximum saponin was extracted. Furthermore, results also revealed that RSM could be successfully used to generate models, optimize the extraction process and predict saponin concentration with the definite ranges for the selected extraction variables. Such established extracted method can be developed for extraction of saponin from other natural sources.

## CRediT authorship contribution statement

**Roza Najjar-Tabrizi:** Formal analysis, Data curation, Investigation, Writing - original draft. **Afshin Javadi:** Formal analysis, Investigation, Writing - original draft. **Anousheh Sharifan:** Software, Funding acquisition. **Kit Wayne Chew:** Validation, Writing - review & editing. **Chyi-How Lay:** Validation, Writing - review & editing. **Pau Loke Show:** Project administration, Resources, Visualization. **Hoda Jafarizadeh-Malmiri:** Conceptualization, Funding acquisition, Methodology, Supervision. **Aydin Berenjian:** Conceptualization, Project administration, Supervision.

## Declaration of Competing Interest

The authors declare that they have no known competing financial interests or personal relationships that could have appeared to influence the work reported in this paper.
